# Simulation of layered soil water transport in the semi-arid region based on Hydrus-3D

**DOI:** 10.1371/journal.pone.0321537

**Published:** 2025-04-15

**Authors:** Yingguo Wang, Haiou Zhang, Yingying Sun, Jian Wang, Lei Shi

**Affiliations:** 1 Shaanxi Provincial Land Engineering Construction Group Co., Ltd, Xi’an, Shaanxi, China; 2 Institute of Land Engineering and Technology, Shaanxi Provincial Land Engineering Construction Group Co., Ltd, Xi’an, Shaanxi, China; 3 Key Laboratory of Degraded and Unused Land Consolidation Engineering, The Ministry of Land and Resources, Xi’an, Shaanxi, China; Kielce University of Technology: Politechnika Swietokrzyska, POLAND

## Abstract

This study addresses the unclear water transport patterns in reconstructed layered soils in the arid and semi-arid climate zones of northwestern China by utilizing the Hydrus-3D model to simulate the rainfall infiltration process. Simulation experiments were designed to investigate different configurations of layered soils, with changes in soil moisture profiles monitored throughout. The water transport characteristics of these soils were comprehensively analyzed from four perspectives: soil moisture, water potential, water flux, and lateral flow within the soil. In order to further explore the influence of interlayer properties on shallow soil moisture dynamics, scenario simulations and global sensitivity analysis were conducted based on optimized models. The results demonstrated that interlayers significantly influence soil water distribution and transport patterns. During the rainy season, soil water content and lateral flow decreased with increasing soil depth, whereas these values increased during the dry season, suggesting that deeper soil layers exhibit strong water storage capacities. Both loess and sandy interlayers impeded water infiltration, albeit through different mechanisms. The loess interlayer retained water due to its low permeability, while the sandy interlayer caused water retention in the overlying clay soil as a result of its low matric potential. Based on the simulation outcomes, it is recommended that a 10 cm thick loess interlayer at a depth of 40 cm in sandy soil enhances upper soil moisture availability for vegetation, whereas a 10 cm thick sandy interlayer at the same depth in loess soil improves soil permeability. This study not only advances understanding of the impact of loess infill on soil moisture dynamics in sandy soil regions but also provides critical guidance for soil reconstruction practices in northwestern China, where sandy soils and loess are predominant.

## Introduction

Soil moisture, a critical variable in the soil-plant-atmosphere continuum, plays a decisive role in the ecological effects of vegetation construction [1]. With the progression of global warming and increased vegetation cover, water scarcity has become increasingly an increasingly pressing issue, and water stress is becoming a major cause of vegetation degradation and mortality, which in turn exacerbates soil degradation [2]. As a core component of the climate system, soil moisture profoundly influences water, energy, and biogeochemical cycles and regulates soil biological and physicochemical processes [3]. Especially in the arid-semi-arid region of northwestern China, where annual precipitation is insufficient and unevenly distributed. In these fragile terrestrial ecosystems, the effective management of soil moisture has become the key to the sustainable development of the ecosystem [4].

Located in arid-semi-arid northwestern China faces the problem of arid climate, shortage of surface water resources, insufficient and uneven distribution of annual precipitation, and extremely fragile terrestrial ecological environment [5–6]. Land reclamation is an important means to protect land resources and improve the regional environment, and soil reconfiguration is the core content of land reclamation, the key lies in soil profile reconfiguration [7]. In the climatic conditions of northwestern China, soil moisture plays a crucial role in soil improvement. It serves as the primary medium for material transport and transformation and is a key limiting factor for sustaining the survival and growth of vegetation and crops. Research has indicated that the laminated structure within the soil significantly reduces the infiltration of soil moisture during the infiltration process [8–11]. Soil reconfiguration for different land use types requires distinct considerations regarding soil water transport. For ecological land use, the focus should be on reducing surface runoff to balance soil evaporation, vegetation water uptake, and groundwater recharge, thereby supporting the long-term stability of hydrological ecosystems. In contrast, for agricultural land use, efforts should prioritize enhancing the water-holding capacity of tillage soil to minimize deep percolation and ensure adequate water availability for crops [12].

For the climatic conditions of soil water in the northwestern part of the country, soil moisture is the primary factor for soil improvement, as it is the carrier of material transport and transformation, and a key constraint for maintaining the survival and growth of vegetation and crops. Studies have shown that the laminated structure in the soil acts as an infiltration reduction for the infiltration process of soil moisture [11–12], which affects the distribution of water. Soil reconfiguration for different land use types has a different emphasis on soil water transport, e.g., for ecological land use, surface runoff should be reduced to balance the relationship between soil evaporation, vegetation water depletion, and groundwater recharge, in order to maintain the long-term stability of hydrological ecosystems [4].

The process of water transport in layered soils is more variable than in homogeneous soils, and the infiltration process of soil water becomes more complicated, mainly due to the sudden change of the hydraulic properties of the soil at the interlayer. Based on variations in soil texture, laminated soil can be categorized into two types: upper coarse and lower fine, and upper fine and lower coarse. However, the mechanisms through which these two structures influence water transport differ significantly. In the case of upper coarse and lower fine soil, due to differing infiltration properties, water encounters hydraulic conductivity obstacles upon reaching the interface [13,14]. Conversely, in upper fine and lower coarse soil, the soil water suction of the upper fine-textured layer prevents water from penetrating into the coarse-textured layer, resulting in the formation of a capillary barrier [15,16]. This capillary barrier is only broken when the water potential of the fine-textured soil at the interface exceeds that of the coarse-textured soil, allowing water flow to continue downward. The location, number, thickness, and texture of the interlayer have a certain effect on the infiltration process of soil water. The infiltration rate becomes constant when the wetting front passes through the interface, and it is proportional to the saturated hydraulic conductivity of the upper soil layer [13]. The higher the layer position of the interlayer, the smaller the corresponding cumulative infiltration [17]; The thickness of the interlayer had the greatest effect on the number of water-holding facies, while the location of the interlayer had the least effect on the number of water-holding facies through the orthogonal test [18].

In summary, the scholars mainly expressed the indicators of wetting front, infiltration amount, and infiltration rate of layered soil in the process of water infiltration, but lacked the revelation of soil water transport mechanism of different structural layered soil profiles. Moreover, for the transition zone between wind-deposited sand and loess in northern Shaanxi, there is an urgent need to put forward scientific and efficient reconstruction technical parameters in the process of soil reconstruction in northern Shaanxi.

In the transition zone between wind-deposited sand and loess in northern Shaanxi, water transport processes in layered soils are significantly influenced by soil texture, structure, and intercalation characteristics. Specifically, we hypothesized:

(1) Hydrological process indicators such as wetting front advance, infiltration volume and infiltration rate of layered soils are not only influenced by the overall soil properties, but also significantly regulated by different structural stratification in the soil profile.
(2) There are differences between loess interlayers and weathered sandstone interlayers in the mechanism of soil water transport. The loess interlayer, because of its poor infiltration performance, mainly plays the role of water insulation, resulting in the retention of soil moisture above the interlayer; whereas the weathered sandstone interlayer, because of its smaller matrix potential and larger air intake value, makes it easier for water to be retained in the relatively fine texture of the soil above the interlayer, thus indirectly blocking the downward transport of water.
(3) The thickness and texture of the interlayer have an important influence on the soil moisture retention. In particular, the thickness of loess interlayer, with the increase of its thickness, significantly increases the blocking effect on soil moisture.

Therefore, this paper intends to carry out numerical simulations of layered soil moisture to reveal the influence mechanism of interlayer soil moisture transport and other related issues, the results of the research in the dry zone layered soil moisture transport theory research and land remediation applications are of great significance.

## Materials and methods

### Test materials

The study area is located in Yuyang District, Yulin City, Shaanxi Province. Yuyang District is located in the eastern part of the Ordos Plateau, which belongs to the typical continental marginal monsoon climate, with four seasons of distinct warm and cold, wet and dry. The average annual precipitation is 365.7 mm and the average annual temperature is 8.3°C (1951–2021). The climate is cold and dry in winter with little rain and snow, and the precipitation is concentrated in summer with many bursts of precipitation.

The experimental site was within the experimental field of Yulin Field Scientific Observatory, Institute of Land Engineering and Technology, Shaanxi Provincial Land Engineering Construction Group Co., with a geographic location of 38.44N, 109.33E. Corn (*Zea mays L.*) is grown on a large scale in the study area and is the main food-producing crop. Most of the areas in the region have a ground-water table depth of 3–10 m [13], and non-homogeneous soil structure is widespread, which is mainly manifested in two forms: natural loess and non-homogeneous body after land remediation. In this paper, the typical sandy soil and loess in the area were taken as the research object, and the physical properties of the two kinds of porous media were obtained by obtaining soil samples from the field and analyzing the particles indoors, as shown in Table 1.

**Table 1:** Physical properties of porous media.

Porous medium	Volume weight/(g cm^−3^)	Particle size composition/%			Texture (USDA)
		Clay (<0.002 mm)	Silt (0.002–0.02 mm)	Sand (0.02–2 mm)	
**Sandy soil**	1.42	8.48	32.60	58.92	sandy loam
**Loess**	1.63	14.46	80.32	5.22	loam

USDA, United States Department of Agriculture [19]

**Fig 1:**
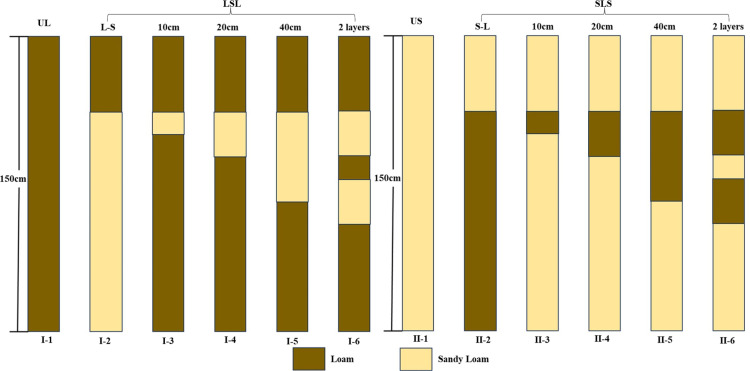
Schematic diagram of the scenario simulation experiment design.

Corn was selected as the research object in this experiment, and irrigation was mainly carried out by atmospheric precipitation. Under the current crop cultivation and economic conditions, a total of 12 experimental plots were set up, each with an area of 1000 cm × 400 cm and a slope of 3°, and the soil texture of each experimental area is shown in Fig 1. Four main treatments were set up in the experiment: homogeneous sandy soil (US), homogeneous loamy soil (UL), wind logged sand-loess-wind logged sand (SLS), and loess-sandy soil-loess (LSL), and each plot was fertilized with conventional fertilizer, and the amount of fertilizer applied was consistent. The plot crops were all corn, and conventional fertilization was used in all plots to ensure the same amount of fertilizer, with 1.0 kg of nitrogen, 1.2 kg of phosphorus, and 0.6 kg of potash applied to each plot.

### Test methodology

The Hydrus-3D model is a finite element model for simulating soil water-salt-thermal transport. Because the atmospheric system has a more complex vorticity representation, the interaction between the atmosphere and the soil medium transport is often ignored in the simulation process. Because the atmospheric system has a more complex vorticity representation, the interaction between the atmosphere and the soil medium transport is often ignored in the simulation process. The model was used to calculate the water absorption of soil roots, and the modified Van Genuchten-Mualem model was used for the soil hydraulic characteristics model [20]. The Feddes model was used to calculate soil root water uptake [21], and the modified Van Genuchten-Mualem model was used to model soil hydraulic parameters.

Assumptions: (1) the experimental soil was homogeneous and isotropic; (2) the effects of soil temperature and the lag effect of soil water quantity and energy relations on soil water movement are ignored. The soil water movement equation is expressed as follows in Eq. (1).


$$\frac{\partial \theta }{\partial t}=\frac{\partial }{\partial x}\left[K\left(\theta \right)\frac{\partial \theta }{\partial x}\right]+\frac{\partial }{\partial y}\left[K\left(\theta \right)\frac{\partial \theta }{\partial y}\right]+\frac{\partial }{\partial z}\left[K\left(\theta \right)\frac{\partial \theta }{\partial z}\right]+K\left(\theta \right)\frac{\partial \theta }{\partial z}-S\left(z,y,z,t\right)$$ (1)

In Eq. (1), Where *θ* is the soil volumetric water content (cm^3 ^cm^−3^); *t* is time (d); *x*, *y* and *z* are spatial coordinates (cm). *K*(*θ*) is the unsaturated soil hydraulic conductivity (cm d^−1^); *S* is the root water uptake (cm d^−1^).

In a homogeneous medium, non-reactive ion transport is simulated by a controlled convective dispersion equation, the mathematical model is given in Eq. (2).


$$\frac{\partial \theta_{c}}{\partial t}=\frac{\partial }{\partial x_{i}}\left(\theta D_{ij}\frac{\partial c}{\partial x_{i}}\right)-\frac{\partial qc}{\partial x_{i}}$$ (2)

In Eq. (2), *i* and *j* represent *x* and *z* axis coordinates, *c* represents solute concentration (g cm^−3^), *q* is the flow rate of water in the soil; *D*_*ij*_ is the hydrodynamic dispersion coefficient.

Root water uptake rate *S*(*h*) represents the volume of water uptaken by the soybean root system from a unit volume of soil per unit of time. It was calculated using the following Feddes model in Eq. (3).


$$S\left(h\right)=\alpha \left(h\right)b\left(h\right)T_{p}$$ (3)

In Eq. (3), where *h* stands for soil matrix potential, and is the water stress response equation. *α*(*h*) is the water stress response equation. *T*_*p*_ is the potential transpiration rate of the crop (cm d^−1^). is the root water uptake distribution function (cm). *b*(*h*) is the root water uptake distribution function (cm^−1^).

The root water uptake distribution function satisfies the condition that


$$\overset{LR}{\underset{0}{\int }}b\left(h\right)dx=1=\overset{M}{\underset{n=1}{\sum }}b_{n}\Delta Z$$ (4)

where *LR* is the thickness of the root zone. *∆Z* is the nodal spacing. *b*_*n*_ is the value of the root distribution function in each spacing, and *M* is the number of nodes occupied by the rhizosphere. Assume that the root system distribution function is linear and has a linear distribution function. Because the main root system of soybean is relatively thick at 0–20 cm, and the thinner it is, the thickness of the root layer in this study is 20 cm, and h takes the value of 0.5 cm. and h takes the value of 0.2.

The initial condition for solving the soil moisture equation of motion is:


$$\theta \left(x,y,z,t\right)=\theta_{0}\left(x,y,z\right),0\leq x\leq X,0\leq y\leq Y,0\leq z\leq Z,t=0$$ (5)

where *θ*_*0*_ (*x*,*y*,*z*) is the initial water content (cm^3 ^cm^−3^), the initial water content of different soil layers is set according to the actual situation, and assumes that the water content of each soil layer into a linear distribution. Content of each soil layer into a linear distribution. *X*, *Y*, and *Z* are the model calculation area’s length, width, and height, respectively. The model takes *X* = 400 cm, *Y* = 1000 cm, *Z* = 150 cm, and the model run time *t* is 365 days.

The upper boundary condition is set as the atmospheric boundary condition, and the lower boundary is set as the free drainage condition. The upper boundary condition is set as the atmospheric boundary condition, and the lower boundary is set as the free drainage condition considering the evaporation from the soil surface. Because the simulation area is large, it is assumed that the moisture movement does not reach the side boundary. The side boundaries are set as the zero flux boundary conditions.

### Model simulation

The model simulates the soil in the depth range of 0–150 cm below the ground surface, using the thickness, location, and number of LSL configurations as the variables of the experiment, and taking into account the root distribution characteristics of the plants in the study area, the thickness of the upper layer of soil was designed to be 40 cm, and the thickness of the interlayer was set to be 10, 20, and 40 cm in increasing order; the number of interlayers was set to be 1 and 2 layers (Fig 1). The scenario experimental design of SLS was the same as that of the LSL. The scenario design of SLS is the same as that of LSL.

The simulation period is from January 1, 2023, to December 31, 2023, a total of 365 d. Variable time-step profiling is used, and the time step is adjusted according to the number of convergence iterations. The initial time step was set as 0.000l d, the minimum step as 0.000l d, and the maximum step as 1 d. The soil water content tolerance was 0.0005, and the pressure head tolerance was 1 cm. After that, the simulation was carried out by segmented and graded methods.

Soil water flow modeling was performed using the Van Genuchten-Mualem model in a single pore model without considering the water hysteresis effect, and the inverse solution method was used to determine the water movement parameters (*Ks*, *θr*, *θs*, *α*, *l*, *n*). The upper boundary of the water flow simulation and the salt simulation were both open atmospheric boundaries, receiving precipitation, irrigation recharge, and evaporation from the soil surface, and the Hydrus water flow simulation was assigned the measured precipitation, irrigation, and evapotranspiration; and the salt simulation was assigned the measured mineralization of the irrigation water. The lower boundary of the water flow simulation is taken as the known variable head boundary, and the pressure head is assigned in Hydrus, which is determined according to the measured depth of the water table; the lower boundary of the salinity simulation is taken as the known concentration boundary, and the measured mineralization of diving is assigned.

The soil hydraulic parameters were based on the measured soil grain size composition, and the initial values of the parameters were given by the automatic generation of the Rosetta program of Hydrus-3D, then the parameters (*q*, *c*, *D*_*ij*_) were fitted by the measured data from the 2023 fertility trial in the test area, and the rate was determined to obtain the initial parameters required for the model simulation. The values of each soil hydraulic parameter in the adjusted Van Genuchten-Mualem equation are given in Table 2.

**Table 2:** Fitting values of hydraulic parameters of VG formula.

Porous medium	θr /(cm^3^ cm^−3^)	θs /(cm^3^ cm^−3^)	*α* /(1 cm^-1^)	*N*	Ks /(cm d^−1^)	*l*
**Sandy soil**	0.0994	0.4762	0.0166	7.6100	0.6596	0.5
**Loess**	0.0420	0.3583	0.0088	1.5544	20.55	0.5

## Results

### Water movement characteristics of layered soils

**Fig 2:**
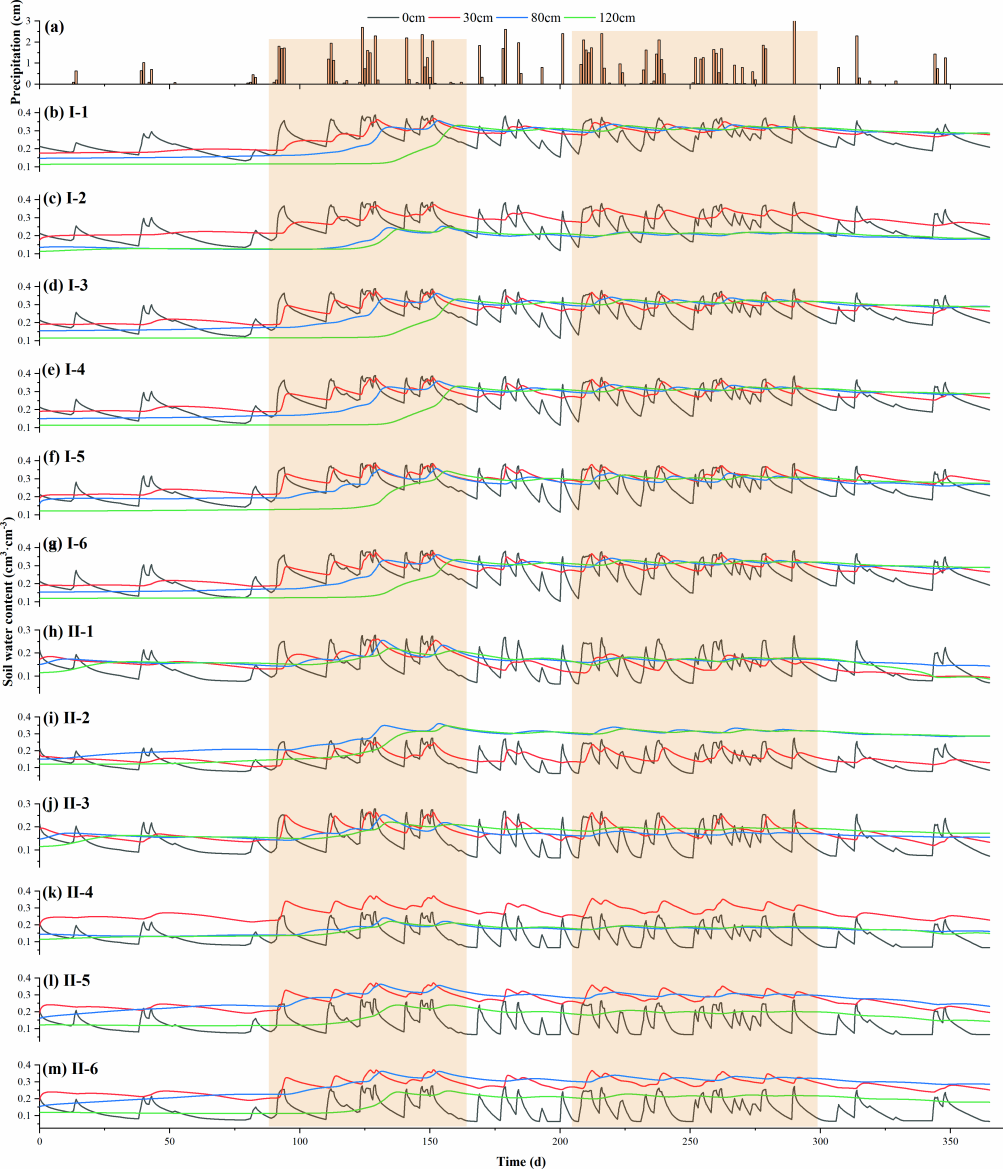
Changes of water content in different depth layers in layered soil structure.

The top layer of soil showed larger fluctuations due to the influence of the water head, while the homogeneous soil (Fig 2b) showed more homogeneous changes overall. During infiltration, the soil moisture in the surface layer is larger, and accompanied by the infiltration of soil moisture, the soil moisture change is stable. The soil water content at the surface layer of the US responded most significantly to rainfall, with fluctuations ranging from 0.12–0.38 cm^3 ^cm^−3^ (Fig 2h), and the fluctuation amplitude increased with the increase of rainfall intensity. After the end of the rainfall, the soil water content declined rapidly, and then slowly declined to the level of the initial state. The degree of response of the soil water content to rainfall at the 30 cm level decreased significantly. The soil moisture content within the 30–150 cm depth range exhibited a significant decrease in response to rainfall. Specifically, the moisture content at the 30 cm depth demonstrated a notable decline, while the moisture content within the 30–150 cm range displayed an apparent lag effect in response to rainfall. The intensity of rainfall was inversely correlated with the lag time, with higher intensities resulting in shorter lag times.

Compared with homogeneous soil, the whole infiltration process of layered soil is more complicated, and its water content changes discontinuously in the layered soil profile. The water content evolution trend of SLS soils from the surface layer to 30 cm depth (Fig 2i–m), which is similar to that of the US, but the fluctuation amplitude is reduced.

For LSL, the soil water content at 0–30 cm showed similar evolutionary patterns to UL, US, and SLS, and the water content in the upper and lower parts of the sand layer was significantly higher than that in the sand layer (Fig 2c–g), mainly because of the incoming suction around the sand layer, and the soil moisture near the interface was able to reach complete saturation, with a larger space for water storage and larger water-resistant barriers. The water content at 80 cm showed an increasing trend, and the higher the rainfall intensity, the faster the water content increased, with an obvious hysteresis effect.

### Soil moisture redistribution in different layered structures

**Fig 3:**
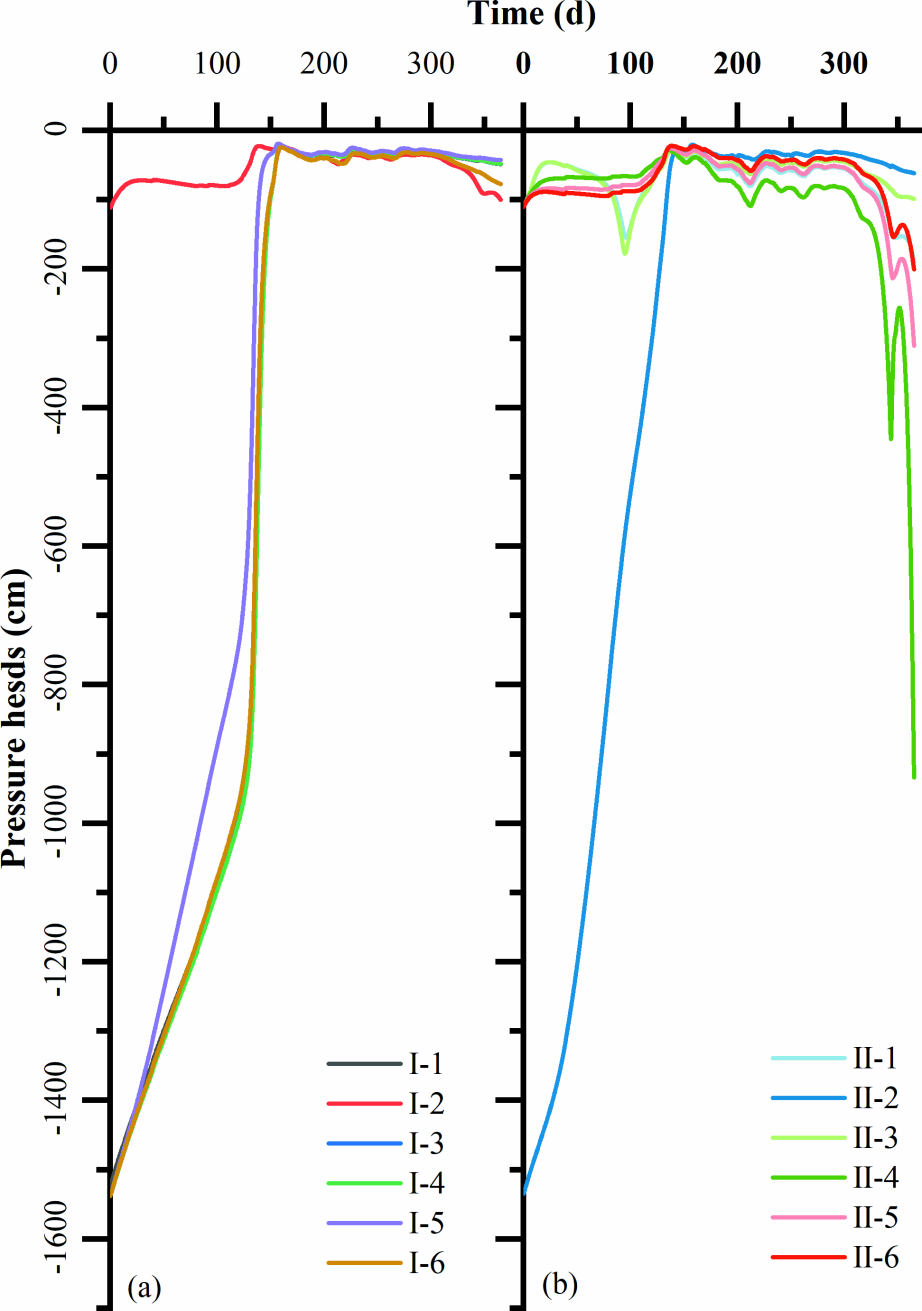
Variation of total water head pressures in layered soil structure over time. **(a)** UL and LSL configuration; **(b)** US and SLS configuration.

Simulation results of water content (Fig 2) and total head (sum of matrix potential and positional potential) (Fig 3) for four soil structures: US, UL, SLS, and LSL. For the US, the soil profile water content was continuously distributed and could be divided into three zones: 0–30 cm was the zone of drastic change, with values fluctuating between 0.08 and 0.37 cm^3 ^cm^−3^; 30–80 cm was the transition zone, where water content changed relatively slowly; and below 80 cm was the stabilization zone, where water content increased linearly with the depth of the soil, and was almost unaffected by the infiltration of rainfall. The total water head of the soil profile showed a smooth and continuous distribution. At the initial moment, the total head of the soil profile was distributed in both dispersive (0–30 cm) and convergent (30–80 cm) patterns. After rainfall, the distribution of the total head of the soil profile shifted to a single convergent pattern, and finally to an infiltration pattern. From the soil profile water flux, it can be seen that after rainfall, the water within 0–30 cm was transported downward, and the water deeper than 30 cm was transported upward, with time, the soil profile water flux gradually decreased, and finally, the water flux of the whole soil profile was downward and decreased linearly with depth.

For the SLS, the water content of the soil profile showed an obvious discontinuous distribution, with a sudden change in the water content at the interface of the interlayer, and the water content within the interlayer was significantly higher than that on both sides of the interlayer; the water content of the soil above the interlayer fluctuated between 0.08 and 0.30 cm^3 ^cm^−3^, especially at 0–30 cm, which was higher than that of the corresponding US location, and the phenomenon of “waterlogging” appeared. In particular, the water content within the 0–30 cm soil layer exceeds that of the corresponding depth in the United States, leading to the phenomenon of “waterlogging.” Conversely, the water content of the soil beneath the interlayer remains unaffected by rainfall infiltration. The total head within the soil profile exhibited a continuous distribution. During rainfall events, only the total head above the mezzanine layer underwent changes. Specifically, the total head at the upper interface of the mezzanine layer exhibited a notable increase, whereas the total head within and below the mezzanine layer remained almost unchanged. Changes in profile moisture fluxes showed that soil moisture above the mezzanine was dominated by downward movement after the occurrence of rainfall, and the soil profile moisture fluxes gradually decreased with time; while the mezzanine and below the mezzanine had almost no moisture fluxes.

For LSL, the overall trend of soil configuration changed gently in the range of 30–150 cm; the soil moisture content also changed at the interface of the mezzanine; the moisture content inside the mezzanine was almost unchanged and lower than that on both sides of the mezzanine; the value and fluctuation of soil moisture content above the mezzanine was smaller than that of the corresponding locations of the US and the SLS; and the moisture content below the mezzanine was slightly reduced. The total water head of the soil profile shifted from a dispersive (0–30 cm) and convergent (30–80 cm) pattern before rainfall to a single convergent pattern after rainfall, and finally to an evaporative distribution. After the occurrence of rainfall, the water in 0–30 cm moved downward, and the water deeper than 30 cm moved upward; with time, all the water in the profile was transported upward, and the water flux decreased with depth.

### Soil interflow fluxes in different layers of soil loam

**Fig 4:**
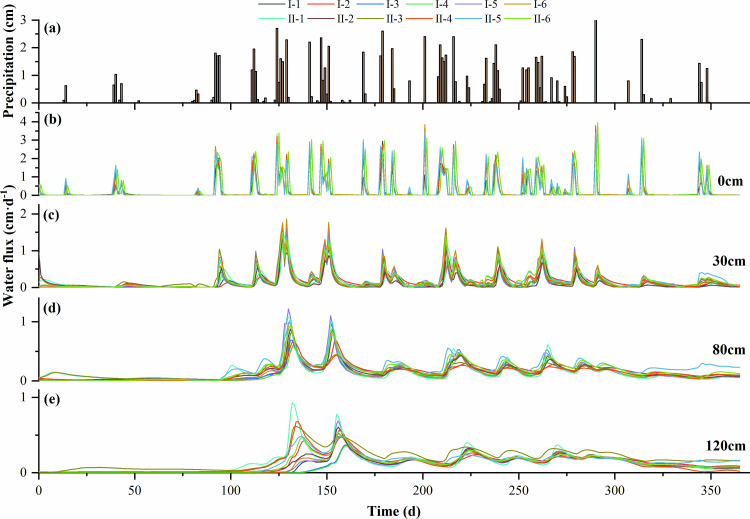
Interflow fluxes in each layer of soil from 0–150 cm of each soil structure. **(a)** 0 cm; **(b)** 30 cm; **(c)** 80 cm; **(d)** 120 cm.

Soil interflow refers to the movement of water within the soil during rainfall, irrigation, and its subsequent period, including infiltration and lateral flow of water within the soil [22]. Lateral flow is an important component of runoff on slopes and has a significant impact on soil moisture and nutrient loss, among others [23]. At the beginning of rainfall, the water transport in the till layer was vertical infiltration. As the rainfall continued, the tillage layer gradually produced lateral flow from top to bottom in the moisture saturation zone (Fig 4). When the water entered the non-tillage layer (>40 cm), the direction of the flow rate in the tillage layer and the non-tillage layer was different, the water in the tillage layer was lateral flow, and the water in the non-tillage layer was mainly vertical infiltration, and the flow fluxes in the tillage loam accounted for the total fluxes, indicating that the loss of water from sloping arable land mainly occurred in the tillage layer (0–40 cm).

**Fig 5:**
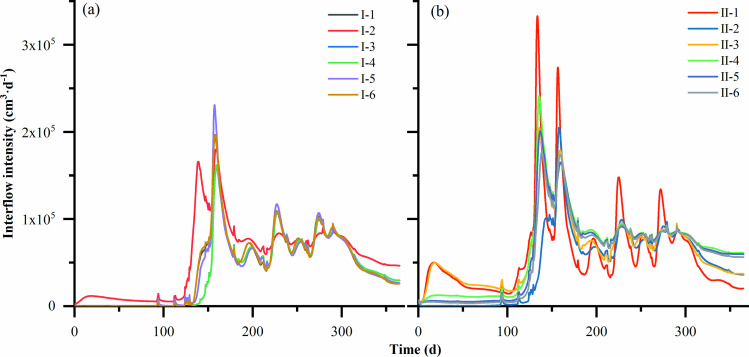
Variation of total soil interflow intensity in each soil structure. **(a)** UL and LSL configuration; **(b)** US and SLS configuration.

The values of SLS and LSL in the same stratum of the same configuration of cropland were close to each other (Fig 5). In the rainy season, the relative change in soil interflow was faster, and there was a corresponding lag effect in the change of soil interflow with the increase of depth. In the dry season, with the decrease in precipitation, the soil water loss was greater than the input, and the deep loam center flow gradually converged, reflecting the corresponding water retention of the soil.

The cumulative soil interflow produced by different layered soil configurations was different (Table 3). The cumulative interflow of loam flow in the UL configuration was 1.46⊆10^7^ cm^3^, in the LSL-10 cm configuration was 1.47⊆10^7^ cm^3^, in the US configuration was 2.36⊆10^7^ cm^3^, and in the SLS-10 cm configuration was 2.29⊆10^7^ cm^3^. The cumulative flow from 0–40 cm in different configurations accounted for more than 60% of the total flux. The effect of soil texture on the soil interflow was more obvious, especially in the 0–20 cm depth. In particular, the abortion flow rate in 0–20 cm depth is about 2–4 times that in other depths.

**Table 3:** Cumulative soil interflow flux with different layered structures.

Test	Cumulative flux/⊆ 10^7^ cm^3^	Test	Cumulative flux/⊆10^7^ cm^3^
Ⅰ**-1**	1.46	**II-1**	2.36
Ⅰ**-2**	2.01	**II-2**	1.75
Ⅰ**-3**	1.47	**II-3**	2.29
Ⅰ**-4**	1.47	**II-4**	2.38
Ⅰ**-5**	1.55	**II-5**	2.22
Ⅰ**-6**	1.50	**II-6**	2.12

## Discussion

### Mechanistic analysis of the blockage of water transport by intercalation

From the experimental and simulation results, it can be found that both loess interlayers and weathered sandy interlayers significantly change the distribution of water potential in the soil profile, and water transport is “blocked” at the interface of the interlayers (Fig 4). The non-homogeneous layered structure had the effect of “storing water and energy” on water transport, and to a certain extent, it filtered the impulsive effects of a single rainfall [24], so that the effects of multiple rainfalls were superimposed, and the water at the interface of the interlayer could only be transported downward when the water overcame the resistance [25]. For the SLS configuration of loamy soil interlayer, the soil shows a blocking effect on soil moisture, when soil water is transported downward, for the loess interlayer, due to its poor infiltration properties, equivalent to the water barrier layer blocking the downward migration of water; for the LSL configuration, weathered sandy soil interlayer, due to the upper part of the fine-grained medium of the water absorption is larger, so that the total soil head above the interlayer is lower than that in the interlayer at the total soil head, causing soil moisture transport was blocked, and the study showed that the greater the rainfall intensity, the stronger the blocking effect, attributed to the greater the rainfall intensity, the stronger the water-driven gas process during rainwater infiltration.

Unlike the law of moisture movement in homogeneous soil, the influence of non-homogeneous soil on moisture movement is mainly due to the heterogeneous layer in the soil body [26], resulting in a water flow blocking effect, which makes the soil pore space at the interface of the interlayer and the hydrodynamic properties of the soil appear obvious changes. The water is retained in the soil layer above the interlayer, thus affecting the transport and distribution of water throughout the entire soil profile, which in turn exacerbates the complexity of the process of precipitation infiltration water flow.

Soil interflow increases with the increase of the average water content in the preliminaries, and there exists an obvious threshold of the average water content in the preliminaries slope for the production of soil interflow, the amount of soil interflow production at the depth of 0–20 cm, is about 2–5 times of the other depths, which is similar to the previous study. The effect of soil moisture content on runoff production and found that the amount of loamy streamflow was small during the dry period, but increased dramatically when the moisture content threshold of 0.45 cm^3 ^cm^−3^ was exceeded [27]. The effect of soil moisture changes on runoff production using a small slope as an example and found that the threshold value of soil moisture was about 0.30 cm^3^cm^−3^ [28]. The threshold values found in different studies varied, which may be related to the differences in topography, soil type, land use, and climatic conditions in the study area [29]. It is also worth noting that soil interflow may also occur when the water content is low in the pre-slope period, provided that the rainfall during that period is very heavy.

Changes in soil interflow were closely related to precipitation, and although there was a time lag characteristic with the occurrence of precipitation events, the flow of soil interflow in sloping cropland tended to rise with increasing precipitation [30–31]. Around April and July, there were more precipitation events and higher soil interflow; in winter, soil interflow occurred 1 day later than precipitation, and in small test plots, the moisture response was faster, indicating that the land was dry in the winter and the soil moisture deficit. Around December-January in winter, although precipitation events also occurred, there was no soil interflow, mainly because of the lower intensity of rainfall in that period and the low initial soil moisture content on the slope, which put the soil moisture in deficit.

In the experimental field, in July–August, when rainfall was predominant, 0–150 cm of loamy mesocosm flowed out basically on the same day in response to rainfall, whereas in March-April and November-December, when rainfall was low, the deeper 80–120 cm was partially fluxed over only on the next day (Fig 5g and h), which further illustrates that the response of loamy mesocosm flow in response to winter moisture deficit needs time. It suggests that soil interflow can be stored in large watersheds, while later acting to nourish soil crops during dry periods.

The Hydrus-3D simulations all showed that the data on water content over time were susceptible to boundary effects. In summary, the LSL configuration stores more water and has a greater depth of infiltration than the SLS configuration of cropland under the same rainfall calendar time and soil conditions.

### Sensitivity analysis of soil hydraulic parameters

To further investigate the influence of key soil hydraulic parameters on water and salt transport in reconstructed layered soils, a global sensitivity analysis (GSA) was conducted using MATLAB 2021b in conjunction with the Hydrus-3D model. The Sobol method, a variance-based GSA approach, was employed to quantify the contributions of individual parameters and their interactions to the variability in model outputs, including soil moisture, water potential, water flux, and lateral flow [32–33].

A total of 1,000 parameter sets were generated using Sobol sequences, ensuring uniform coverage of the parameter space. Each parameter (*θr*, *θs*, *Ks*, *α*, *N*) set was then input into the Hydrus-3D model to simulate the rainfall infiltration process under the same boundary conditions as described in the study. The model outputs, including soil moisture profiles and lateral flow rates, were recorded for each simulation.

**Fig 6:**
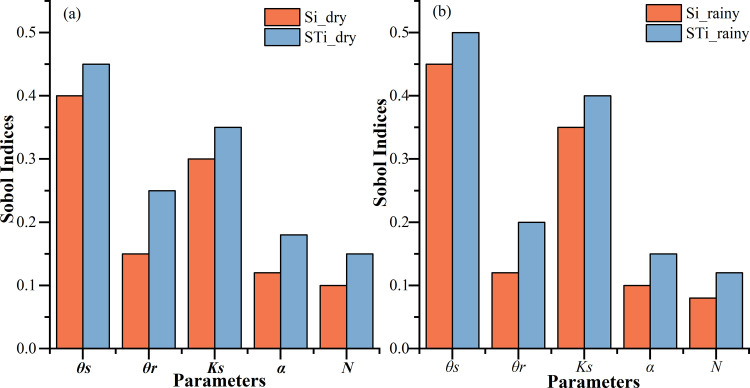
Global sensitivity analysis results of soil hydraulic parameters under different conditions. **(a)** Dry season; **(b)** rainy season.

MATLAB was utilized to compute the Sobol indices, encompassing both the first-order (main effects) and total-effect indices. The first-order indices (Si) measure the individual contribution of each parameter to the output variance, whereas the total-effect indices (STi) encompass both individual and interactive effects. According to the sensitivity analysis (Fig 6), the value of *θs* emerged as the parameter with the highest first-order sensitivity index (Si ≈ 0.45) for soil moisture dynamics, predominantly affected lateral flow dynamics, particularly in the presence of interlayers, where it modulated the soil’s water storage capacity. The value of *Ks* emphasizing its pivotal role in controlling water infiltration and redistribution within the soil (Si ≈ 0.35). The value of α significantly influenced the distribution of water potential (Si ≈ 0.30), particularly in the upper soil layers, affecting water retention and release rates. Conversely, the value of *θr* and *N* had relatively smaller individual contributions (Si < 0.15), but showed notable interactive effects, as indicated by their total-effect indices (STi ≈ 0.25 and 0.20, respectively). In summary, the sensitivity of soil moisture infiltration at different depths to various parameters is ranked from high to low as *θs*>*Ks*>*θr*>α >*N*.

Based on the GSA results, the sensitivity analysis underscores the critical role of *θs* and *Ks* in governing water transport patterns in reconstructed layered soils. These findings align with the study’s experimental results, which highlighted the importance of interlayer properties in regulating soil moisture dynamics. Specifically, the low permeability of loess interlayers and the low matric potential of sandy interlayers were identified as key mechanisms influencing water retention and redistribution.

### Ecological significance of layered soil water retention

The natural vegetation in the inland Northwest has a strong dependence on soil water, while the lithologic structure of the air-packed belt has an obvious influence on the distribution of soil water, and the combined structure of multiple lithologies has a larger effective water-holding capacity and stronger ecological effect compared with a single lithology.

The blocking effect of layered soil on water transport can provide a basis for ecological restoration of vegetation and water resource management in dry areas. For example, in the wind-sand beach land of Maowusu, the strong potential evaporation, leads to scarcity of precipitation. Most precipitation events are of low intensity, causing the infiltrated water to be lost through evaporation before it can reach deeper soil layers, thereby rendering it ineffective. In contrast, during intense precipitation, the rainwater infiltrates rapidly into deeper soil layers, resulting in leakage and preventing it from being effectively retained in the shallow soil for vegetation use. It is believed that the soil structure can be reconstructed through the windy sandy beach land, the clay layer is set at a certain depth, and the layered soil structure is constructed, so that when the soil water is transported downward, due to the poor infiltration performance of the fine particles, the water transport speed will be slowed down, thus strengthening the water stagnation effect of the envelope, and the effective water held in the envelope can be supplied to the vegetation, and give play to the ecological function of the water in the shallow air envelope [33], and at the same time, it can inhibit evapotranspiration by interrupting the capillary rise height to inhibit evaporation [34–35]. In addition, in the process of ecological restoration and land reclamation, the soil environment can be improved and plant growth can be promoted by installing interlayers to improve the distribution characteristics and transportation law of soil moisture.

There are many factors affecting water transport in layered soils, and the texture and stratification order of layered soils have significant effects on rainfall infiltration processes and soil water redistribution. Different soil layer sequences caused significant differences in cumulative infiltration amount and infiltration rate [36]. The higher the coarse sandy soil, the greater the cumulative infiltration volume and infiltration rate in the bordering areas of Jin, Shaanxi, and Mongolia [13]. The root system of most dominant vegetation in northern Shaanxi is mainly distributed in the soil layer shallower than 40 cm, and the soil water in this range is also the main source of root water consumption [28,37]. The design of a sandwich-type soil profile for Yellow River sediment-filled reclamation based on the effects of different thicknesses, locations, and number of interlayers on the infiltration and evaporation characteristics of reconstructed soils [38]. Soil configuration in arid zone affects water movement mainly due to the water flow blocking effect caused by heterogeneous layers in the soil [39]. In this study, based on the characteristics of loess interlayer on the reconstruction of soil water infiltration blockage, we simulated the effect of different thicknesses and number of interlayers on the soil water storage above the interlayer when the location of the upper interface of the interlayer is at 40 cm, and obtained the results of the simulation results of the pre-analysis that the soil water infiltration is insensitive to the number of interlayer, and logarithmic growth relationship with the thickness of the interlayer.

As the thickness of the sandwich increases, the soil water storage above the sandwich increases in a logarithmic function (Fig 5). When the thickness of the interlayer is between 0 and 20 cm, the soil water storage is in a rapid increase stage; when the thickness of the interlayer exceeds 20 cm, the trend of increasing soil water storage above the interlayer slows down gradually. The infiltrated rainwater is stored in the overlying fine layer, and with the increase of infiltration, the water accumulates at the interface, the suction at the interface decreases gradually, and when the suction is small enough, the water breaks through from the fine layer to the coarse layer, and seepage occurs [29,40]. In a certain thickness range, with the increase of the thickness of the viscous interlayer, the resistance of the water-driven gas becomes larger, the less likely the water is to infiltrate downward; when the viscous interlayer reaches a certain thickness, the resistance is close to the maximum value, and is no longer affected by the thickness [41–42]. It also means that laying a loess interlayer with a thickness of about 10 cm at a depth of 40 cm in wind-deposited sandy soil can improve the effective moisture of shallow soil while setting a sandy interlayer with a thickness of 10 cm at a depth of 40 cm in loess is conducive to maintaining the permeability of the soil, and facilitates the absorption of the root system of vegetation, and thus can be used as a technical parameter of soil improvement to improve the survival rate of vegetation. The results of this study echo these international studies and further confirm the important role of layered soil structure in soil water transport and land restoration practices. For the arid and semi-arid region of Northwest China, which is distributed with a vast loess area, the constructed soil structure not only matches the actual situation of the study area, but also provides a useful reference for ecological restoration and water resource management in arid and semi-arid regions around the globe.

## Conclusions

The water content of the layered soil profile showed discontinuous characteristics, with abrupt changes observed at the intercalation zones. In contrast, the soil profile’s negative pressure was distributed continuously, and rainfall events only influenced the matrix potential of the soil profile above the intercalation. The magnitude of this change was positively correlated with the intensity of the rainfall. However, the mechanisms governing soil moisture transport differed between loess interlayers and sandy interlayers. Specifically, the clayey interlayer in loess soil exhibited poor infiltration properties, leading to water retention in the soil above the interlayer. Conversely, in sandy soil, the small matrix potential and large air entry value of the coarse-phase interlayer resulted in water retention in the relatively fine-textured soil above the interlayer.

The effects of soil texture and structure on soil moisture transport were investigated. The sensitivity analysis underscores the critical role of *θs* and *Ks* in governing water transport patterns in reconstructed layered soils. For loess interlayer, the poorer the infiltration performance, the stronger the water barrier effect, while for sandy soil interlayer, it is indirectly by increasing the upward transmission of water to block the downward transmission of water, the two kinds of structure of the soil water blocking effect of loess interlayer performance is outstanding. The thickness of the loess interlayer has a significant effect on the soil water retention effect, with the increase of interlayer thickness, the retention effect is enhanced.

From the perspective of enhancing the effectiveness of shallow soil water, it is concluded that setting a 10-cm-thick loess interlayer at a depth of 40 cm in sandy soil and setting a 10-cm-thick sand interlayer at a depth of 40 cm in loess is an ideal technical parameter of soil reconstruction, which can improve the water retention of soils above the interlayer and give full play to the role of “soil reservoir” to meet the ecological water demand of vegetation in dry areas.

Although the study of layered soil profiles revealed the different effects of loess and sandy interlayers on soil moisture transport and water retention, and resulted in technical parameters to optimize soil moisture effectiveness, there are still some issues that need to be further explored and resolved. However, there are still some issues that need to be further explored and resolved, such as whether these technical parameters are still applicable under different climatic and soil conditions; the long-term stability of loess and sandy interlayers and their effects on the soil environment; and how to ensure the accuracy and uniformity of the interlayer setup in practice; and the need to carry out long-term monitoring.

## Supporting information

S1 Table Rainfall in 2023 in Yuyang District, Yulin City, China. (XLSX)
